# IMRA/SRS Delphi consensus on international standards for common core components of robotic surgical training design

**DOI:** 10.1007/s11701-024-02057-8

**Published:** 2024-09-19

**Authors:** Jessica Wynn, Anthony Costello, Kirsten Larkins, Daniel Costello, Ahmed Ghazi, Kieran Ryan, Kevin Barry, Matthew Gray, Anthony Gallagher, Andrew Hung, Alexander Heriot, Satish Warrier, Farleigh Reeves, Justin Collins, Phil Dundee, Justin Peters, David Homewood, Dean Driscoll, Owen Niall, Tayla Fay, Ajit Sachdeva, Henry Woo, Richard Satava, Helen Mohan

**Affiliations:** 1 International Medical Robotics Academy (IMRA), Melbourne, VIC Australia; 2 Department of Surgery, Peter MacCallum Cancer Centre, Melbourne, VIC Australia; 3 Department of Surgery, University of Melbourne, Melbourne, VIC Australia; 4 Department of Urology, Royal Melbourne Hospital, Melbourne, VIC Australia; 5 Department of Urology, John Hopkins Hospital, Baltimore, MD USA; 6 Royal College of Surgeons in Ireland, Dublin, Ireland; 7 ORSI Academy, Melle, Belgium; 8 Department of Urology, Cedars-Sinai Medical Centre, Los Angeles, CA USA; 9 Department of Urology, University College London Hospital, London, England; 10 Department of Urology, St. Vincent’s Hospital Melbourne, Melbourne, VIC Australia; 11 University of Washington, Seattle, WA USA; 12 Royal Australasian College of Surgeons, Melbourne, VIC Australia

**Keywords:** Robotic surgery, Curriculum, Training, Consensus

## Abstract

Robotic surgery has expanded internationally at pace. There are multiple local robotic training pathways but there is inconsistency in standardisation of core common components for curricula internationally. A framework is required to define key objectives that can be implemented across robotic training ecosystems. This Delphi consensus aimed to provide recommendations for core considerations in robotic training design across diverse training environments internationally. A literature search was performed and an international steering committee (AG, KL, JW, HM, TC) proposed key components for contemporary robotic training design and a modified Delphi approach was used to gather stakeholder opinion. The outcomes were then discussed at a face-to-face international expert consensus at the IMRA educational session at the Society of Robotic Surgery (SRS) meeting and final voting was conducted on outstanding items. Stakeholders included robotic surgeons, proctors, trainees and robotic surgical training providers. There was consensus achieved in 139 statements organised into 15 themes. There was 100% agreement that standardised themes in robotic curricula may improve patient safety. Key take-home messages include—training curricula should be multiplatform, non-technical skills are an important component of a robotic curriculum as well as console and bedside skills, clinically relevant performance metrics should be used for assessment where available, the reliance on cadaveric and live animal models should be reduced as high-fidelity synthetic models emerge, and stepwise component training is useful for advanced procedural training. These consensus recommendations are intended to guide design of fit for purpose contemporary robotic surgical curricula. Integration of these components into robotic training pathways internationally is recommended.

## Introduction

Robotic assisted surgery (RAS) has over the last two decades expanded in its applications and is increasingly accepted globally to provide minimally invasive surgery, delivering improved patient outcomes in many settings [1–3]. RAS is now utilised across multiple surgical specialties, including urology, general surgery, paediatric surgery, gynaecology, cardiothoracic surgery, and otorhinolaryngology [3]. Training has struggled to keep pace with the widespread adoption of robotic surgery, especially as access to robotic platforms during surgical training is limited. Increasingly, surgeons and trainees are likely to encounter more than one robotic platform, adding additional impetus for international standards for robotic training. While the initial early adopters of robotics were established surgeons adapting to robotic surgery, there is now increasingly a need to train robotic surgeons as part of their standard surgical training pathway so they are appropriately skilled when they enter independent practice.

A RAS curriculum should look to incorporate the needs of the trainee, the views of the trainer and the relevant regulatory bodies [1]. The current literature discussing development of a standardised robotic curriculum does not account for adaptation internationally. Pre-existing RAS curriculum include linear trajectories of online learning, device training, simulation followed by procedural training [2–4]. There is an emphasis on mentorship from qualified surgeons [3] and focus on the assessment of skills and performance prior to operating on real patients [5].

There is currently a mismatch between the access to RAS training and desire for training opportunities globally, despite there being a need for surgeons emerging into the workforce increasingly to be trained in robotic surgery. Surgical trainees are keen to have access to robotic training [6, 7]. There are local examples of robotic training pathways [8], but issues like access and scalability remain. There are also speciality specific or procedure specific examples in robotic curriculum development. Overall, there is considerable variability in current robotic training pathways. Several authors have called for a standardisation of international RAS curricula [1–3, 5]. These to date have also not considered the emergence of novel platforms and their impact on training needs or training design.

There are multiple stakeholders in considering a robotic training curriculum from trainees to trainers, Royal colleges, robotic training organisations and robotic vendors and different models of delivery of robotic training that have evolved internationally. A challenge to developing a RAS curriculum is the need to consider evolving robotic technology and the multiple platforms now available to use in RAS [3]. Understanding multiplatform learning is likely to impact on future curricular design [9]. This Delphi was conducted to achieve consensus on the core components that all international robotic training pathways should include in structured robotic training.

Establishing internationally agreed principles on robotic curriculum design is needed, ensuring that curricula are fit for purpose internationally. This Delphi aimed to achieve consensus for objectives, implementation, and certification in robotic surgical training design adaptable to diverse training ecosystems.

## Methods

This project was carried out in four phases: a review was conducted of current literature on a curriculum in robotic training. A steering group (HM, JW, KL, AG, TC, DC, DD) was formed to discuss the current literature and develop an online Delphi questionnaire. The Delphi voting was conducted by three rounds of online review and voting. Any statement that reached 80% consensus was included in the final document. A face-to-face consensus session was held any areas of debate discussed and voted on and the consensus statement completed.

### Review

A review of pre-existing consensus and guidelines on a robotic curriculum was performed following the Preferred Reporting Items for Systematic Reviews and Meta-analysis guidelines (PRISMA). In August 2023, a computerised search was conducted on PubMed and Medline databases. The search utilised medical subject headings included “consensus” or “guideline”, “robotic surgery training” and “curriculum”. All article types were included in abstract screening and there were no parameters set on date published. Only papers written in English were included. The search yielded 143 papers which underwent abstract review, and if applicable, full text review. An additional paper was found on grey literature search. A total of nine papers were included in the review, five were consensus papers specific to the development a robotic surgery training curriculum more broadly and three were specific for sub-specialty training programs. From these publications previously examined curriculum components were identified, or gaps in the current curriculum design recommendations were highlighted.

### Steering committee

A steering committee discussed the literature and current outstanding questions for development of a structured robotic training programme with common core components internationally. The steering committee was composed of robotic surgeons (A Ghazi, H Mohan, T Costello), surgical trainees (J Wynn, K Larkins, D Costello) and educational curriculum developers (D Driscoll). This was performed as an online meeting with subsequent agreement on final questions via email. An online first round of a Delphi questionnaire was generated and circulated using SurveyMonkey (Supplementary Table 1).

### Online Delphi rounds

The survey was circulated via email to 24 participants including qualified surgeons, other healthcare professionals, academics, simulation experts from aviation, curriculum developers and surgical trainees. A three-round Delphi process was used to extract expert consensus. Respondents were required to answer “true” or “false” to a series of statements. There was opportunity at the end of each section to provide free-text feedback on additional questions that should be included. Voting was conducted anonymously. Questions which reached 80% or greater consensus were removed prior to the next round of the survey. Questions were developed from free-text feedback and included in the next round of the survey. Voting in subsequent in rounds was informed by knowledge of results from the previous round, displayed as percentages alongside each question.

### Expert consensus at Society of Robotic Surgeons (SRS) conference

A dedicated live panel discussion formed part of the IMRA robotic education session at the Society of Robotic Surgeons (SRS) international robotic meeting in Melbourne July 2023. It was delivered alongside presentations which contextualised the current state of robotic surgery education worldwide and included open discussion inviting audience participation. A nine-member panel was formed to discussed survey topics and free-text responses which led to contentious responses amongst survey respondents. The panel was made up of qualified surgeons, healthcare professionals, academics, and members of the aviation industry. This consensus was recorded for analysis.

Topics discussed included solutions to train for a multi-platform world, multi-level learners and adaptive training, training to proficiency versus competency, meaningful assessment and feedback for learning, bridging the gap between pre-clinical and clinical learning and ensuring equity and diversity in access to robotic training.

#### Thematic content analysis

Transcripts from this panel discussion were analysed using NVIVO and principles of thematic analysis as described by Braun and Clarke [10].

## Results

### Literature review

**Fig. 1:**
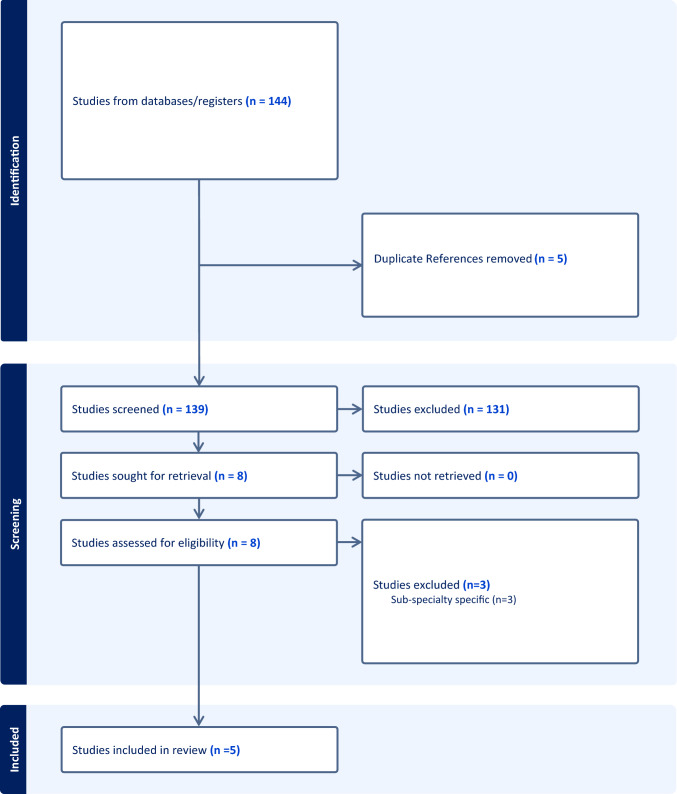
PRISMA diagram literature review

A systematic review into whether there was pre-existing consensus on the development of a standardised robotic curriculum was performed following the Preferred Reporting Items for Systematic Reviews and Meta-analysis guidelines (PRISMA). In August 2023, a computerised search was conducted on PubMed and Medline databases. The search utilised medical subject headings including “consensus” or “guideline”, “robotic surgery training” and “curriculum”. All article types written in English were included in abstract screening. There were no publishing date parameters set. The database search yielded 143 papers and an additional paper was found on grey literature search. Eight papers were included in the full text analysis. Three were excluded as they were sub-specialty specific. The remaining five papers were included for data extraction. The PRISMA results are included in Fig. 1.

Five papers published between 2008 and 2023 were included. Two were published by American groups, one by a Dutch group, one by a group based in United Kingdom and Ireland and one article described consensus opinion from a group of international experts. Although the respondents to the surveys distributed in these consensus articles were varied, with Burke et al. 2023 focussed on trainee insights only, and other articles focussed on the mixed opinions of faculty and trainees, all papers agreed on the importance of a standardised curriculum developed for the needs of the learner [1–3, 5, 11]. All articles encouraged simulated learning and reinforcement of skills outside of the operating theatre before they were put to practice. All articles advocated for objective metric-based assessment of the learner and clear certification pathways to ensure patient safety. Ahmed et al. (2015) reported agreement among international experts that there needed to be additional training for the trainer. All articles acknowledge access to the technology being a barrier to trainees accessing robotic education. No identified article focused on the design of a robotic curriculum for diverse training ecosystems.

### Steering committee themes

On discussion of the gaps in the literature with the steering committee from these five papers, developed questions across 15 aspects important in the development of framework to guide curriculum design in robotic surgical training. Questions generated by steering committee are included in appendix 1.

### Delphi online voting

There was an 83.3% (20/24) response rate in the first online round, 75% (18/24) response rate in the second and 66.7% (16/24) response rate in the final online round. Consensus statements are included in Table 1.

**Table 1:** Items that achieved consensus

Question	Yes	No	Round consensus achieved
Role of a robotic curriculum			
Standardised themes in an international robotic training curriculum is important for resource consolidation	94.44%	5.56%	1
Standardised themes in an international robotic training curriculum is important to establish a large international database of robotic surgery	94.44%	5.56%	1
Standardised themes in an international robotic training curriculum is important in developing international collaborations in robotic education	94.44%	5.56%	1
Standardised themes in an international robotic training curriculum may improve patient safety	100.00%	0.00%	1
The lack of a standardised international curriculum for robotic training is a risk to patient safety	88.24%	11.76%	2
The lack of a standardised international curriculum for robotic training is a barrier to robotic training	88.24%	11.76%	2
The lack of a standardised international curriculum for robotic training is a barrier to trainees and fellows accessing quality robotic training	88.24%	11.76%	2
A standardised robotic training curriculum may allow development of common processes in training robotic surgeons	100.00%	0.00%	1
A standardised robotic training curriculum may allow development of standardised evaluation of robotic competency (displaying minimum skill to safely accomplish a task)	100.00%	0.00%	1
Core components of an international robotic curriculum			
An international robotic curriculum would include a stepwise training pathway	100.00%	0.00%	1
An international robotic curriculum would be modular with basic to advanced general to specialty specific modules	100.00%	0.00%	1
An international robotic curriculum should be multimodal – e.g. include online learning, laboratory simulation and operating on patients	100.00%	0.00%	1
An international robotic curriculum should include guidance on training when operating on patients	100.00%	0.00%	1
Q29. Do you think this curriculum would be different for novice robotic surgeons regardless of prior non-robotic surgical experience (i.e. residents, consultants, fellows, etc.)?	88.24%	11.76%	2
Flexibility of an international curriculum			
An international robotic curriculum would be modifiable to suit each region/country	94.44%	5.56%	1
An international robotic curriculum would be modifiable by each institution	88.24%	11.76%	2
An international robotic curriculum should provide guidance rather than being prescriptive	83.33%	16.67%	1
An international robotic curriculum should evolve over time as new knowledge on robotic training emerges	100.00%	0.00%	1
Educational goals of an international curriculum			
Educating to proficiency (consistent demonstration of a high degree of performance) should be a goal of an international robotic surgery curriculum	94.44%	5.56%	1
The goal of an international robotic surgery curriculum should be to provide an introduction to robotic surgery	82.35%	17.65%	2
An international robotic curriculum should include awareness of multi-console learning	88.89%	11.11%	1
An international robotic curriculum should include non-technical skills learning outcomes	100.00%	0.00%	1
An international robotic curriculum should include covering the risks of robotic surgery	100.00%	0.00%	1
An international robotic curriculum should aim to make surgeons aware to trouble shoot robotic surgery pitfalls	100.00%	0.00%	1
An international robotic surgery curriculum should also be developed for the wider surgical team	94.44%	5.56%	1
Achieving competence (the display of minimum skill to safely accomplish a task) in robotic assisting should be a learning objective of an international robotic curriculum	88.89%	11.11%	1
Achieving competence (the display of minimum skill to safely accomplish a task) in robotic console operating should be a learning objective of an international robotic curriculum	94.44%	5.56%	1
An international robotic curriculum should provide a basic introduction to concepts in robotic surgery	88.89%	11.11%	1
An international robotic curriculum should provide a roadmap to achieve robotic competency (c)	94.44%	5.56%	1
An international robotic curriculum should aim for proficiency (consistent demonstration of a high degree of performance) rather than just competency (displaying minimum skill to safely accomplish a task)	83.33%	16.67%	1
An international robotic curriculum should fit multiple robotic disciplines and specialties	100.00%	0.00%	1
Basic didactic pre-device training			
Do you think basic didactic training is valuable for robotic training?	100.00%	0.00%	1
Should it be delivered as a prerequisite to progressing to device training?	83.33%	16.67%	1
Could basic didactic pre-device training be delivered in parallel to device training?	88.24%	11.76%	2
Should it be completed prior to operating on patients?	100.00%	0.00%	1
Should it be generic cross specialty basic robotic training?	83.33%	16.67%	1
Should it cover basic robotic setup?	100.00%	0.00%	1
Should it include how to troubleshoot e.g. if instrument stuck?	100.00%	0.00%	1
Should it be multi-platform?	94.44%	5.56%	1
Should it be modular with the option of adding additional platforms?	100.00%	0.00%	1
Should basic didactic pre-device training be delivered online?	94.12%	5.88%	2
Should it be hybrid online and in person?	100.00%	0.00%	1
Should the learner complete basic didactic pre-device training independently?	100.00%	0.00%	2
Should the learner complete basic didactic pre-device training with other learners?	82.35%	17.65%	2
Should basic didactic learning be assessed?	88.89%	11.11%	1
Online learning is an important component of an international robotic curriculum	100.00%	0.00%	1
Didactic learning should include learning about hardware in a robotic console	100.00%	0.00%	1
Didactic learning should include learning about hardware in a robotic cart-side	94.44%	5.56%	1
Should docking and undocking be included in basic didactic learning?	100.00%	0.00%	1
Should familiarity with different robotic instruments be included in basic didactic learning	100.00%	0.00%	1
Device training			
Should hands-on console training be included in an international robotic curriculum?	100.00%	0.00%	1
Should hands-on patient-side cart training be included in an international robotic curriculum?	100.00%	0.00%	1
Basic robotic device training should include hands-on learning about hardware in a robotic console	94.44%	5.56%	1
Basic robotic device training should include hands on learning about hardware in a robotic patient-side cart	94.44%	5.56%	1
Should docking and undocking be included in basic robotic device orientation?	100.00%	0.00%	1
Should familiarity with different robotic instruments be included in basic robotic device orientation?	94.44%	5.56%	1
Should docking and undocking be included in a basic robotic device orientation?	100.00%	0.00%	1
Basic console skills simulation			
Basic console simulation should include virtual reality simulation e.g. using simulation modules?	100.00%	0.00%	1
Basic console simulation should be split into two parts- virtual reality and laboratory using models?	83.33%	16.67%	1
Simulation using a box style training with synthetic tissue models or tissue should be included as part of a robotic training curriculum	100.00%	0.00%	1
Retraction using the 4th arm is a core component of robotic basic skills simulation	94.44%	5.56%	1
Control of robotic instruments is a core component of a robotic training basic skills simulation	100.00%	0.00%	1
Depth perception is a core component of a robotic training basic skills simulation	88.89%	11.11%	1
Suturing is a core component of a robotic basic skills simulation	100.00%	0.00%	1
Cutting is a core component of a robotic basic skills simulation (e.g. cutting out a shape)	100.00%	0.00%	1
Camera control is a core component of a robotic basic skills simulation	100.00%	0.00%	1
Are virtual reality simulators useful for mastering these skills?	88.89%	11.11%	1
Should basic robotic skills training be multi-platform?	83.33%	16.67%	1
Should there be a modular option to train in different platforms?	100.00%	0.00%	1
Completion of basic robotic training should include an evaluation of competency (displaying minimum skill to safely accomplish a task)	100.00%	0.00%	1
			1
Advanced didactic training			
Procedure specific didactic training should be a component of an international robotic training curriculum?	88.89%	11.11%	1
Procedure specific didactic training should be performed in parallel to procedural simulation training?	100.00%	0.00%	3
Procedure specific didactic training should be performed as a prerequisite to procedural simulation training?	83.33%	16.67%	1
Procedure specific didactic training should be performed prior to operating on patients?	94.44%	5.56%	1
Procedure specific didactic training should include one main method for performing a procedure	88.24%	11.76%	2
Procedure specific didactic robotic training should include variations in methods of performing a procedure	88.24%	11.76%	2
Procedure specific robotic training should include some education about complications	100.00%	0.00%	1
Procedure specific didactic robotic training should be assessed	100.00%	0.00%	1
Satisfactory outcome in the evaluation of procedure specific didactic robotic training should be a prerequisite to progress to further laboratory simulation	88.89%	11.11%	1
Satisfactory outcomes in the evaluation of procedure specific didactic robotic training should be a prerequisite to operating on humans	94.44%	5.56%	1
An evidence-based approach should be taken to pre procedure robotic training	100.00%	0.00%	1
Advanced procedural training			
Stepwise component simulation can be a useful method for advanced procedural training	100.00%	0.00%	1
Whole procedure training provides optimum training for advanced procedural training	92.86%	7.14%	3
High fidelity models (e.g. high fidelity synthetic models or cadaveric or tissue models) are an important component of advanced procedural training	100.00%	0.00%	1
Models should incorporate clinically relevant performance metrics where possible	100.00%	0.00%	1
Performance in procedural models should be to competency (displaying minimum skill to safely accomplish a task)	82.35%	17.65%	2
Performance in procedural models should be to proficiency (consistent demonstration of a high degree of performance)	83.33%	16.67%	1
Models for training			
Synthetic models e.g. hydrogels are an acceptable alternative to cadaveric or animal models for basic training	100.00%	0.00%	1
The reliance on cadaveric and live animal models should be reduced as high-fidelity synthetic models emerge	100.00%	0.00%	1
High fidelity synthetic or hydrogel models can provide an alternative to tissue-based simulation for procedural skills	88.89%	11.11%	1
“Dissectability” is a core component of a model for advanced robotic training	100.00%	0.00%	1
Ability to cauterise is a core component of a model for advanced robotic training	100.00%	0.00%	1
Ability to replicate procedural steps is an important component of a model for advanced robotic training	100.00%	0.00%	1
Accessibility is a barrier	100.00%	0.00%	1
The exact model used for training is not as important as the functionality of the model, e.g. if it can be appropriately dissected, cut, stapled and cauterised etc. and provide appropriate fidelity simulation	100.00%	0.00%	3
Operation room training			
The curriculum should include criteria to determine if a learner is ready to progress onto conducting procedures on patients?	100.00%	0.00%	1
The criteria to progress onto operating on patients is different for a consultant aiming to perform robotic procedures compared to a trainee who is being directly supervised to do components under supervision	88.24%	11.76%	2
Should simulation training be ongoing once a learner is operating on patients?	94.12%	5.88%	2
Standardised evaluation tools e.g. GEARS should be used by proctors or supervisors when assessing performance when operating on patients	83.33%	16.67%	1
Clinically relevant performance metrics should be utilised where available	100.00%	0.00%	1
Multilevel learning should be considered in a curriculum e.g. consultants, fellows and trainees at different stages on the learning curve	100.00%	0.00%	1
A robotic curriculum would help improve robotic fellowship training	100.00%	0.00%	1
A minimum number of supervised cases should be performed	83.33%	16.67%	1
The number of cases to reach competency (displaying minimum skill to safely accomplish a task) may vary	100.00%	0.00%	1
“Sign off” should be based on competency (displaying minimum skill to safely accomplish a task) rather than case numbers	94.44%	5.56%	1
Non-technical skills			
Non-technical skills should be a component of didactic learning	100.00%	0.00%	1
Non-technical skills should be simulated during simulation training	100.00%	0.00%	1
Non-technical skills should be included in operating room training	100.00%	0.00%	1
Non-technical skills should be assessed in didactic learning	83.33%	16.67%	1
Non-technical skills should be assessed during simulation training	100.00%	0.00%	1
Non-technical skills should be assessed in operating room training	100.00%	0.00%	1
Awareness of startle effect (the physical and mental response to a sudden unexpected stimulus) should be a component of non-technical skill simulation	88.89%	11.11%	1
Emergency undocking should be taught as part of non-technical skills training	100.00%	0.00%	1
Managing the surgical team in a robotic theatre is an important component of non-technical skills training	100.00%	0.00%	1
Managing communication in robotic surgery is an important component of non-technical skills training	100.00%	0.00%	1
Implementation of a robotic curriculum			
Accessing robotic training is currently limited by access to robotic training facilities	94.44%	5.56%	1
Accessing robotic training is currently limited by access to robotic platforms	94.44%	5.56%	1
Consideration to equity in surgical training is required in providing robotic training opportunities	94.44%	5.56%	1
Cost is a barrier to robotic training	94.44%	5.56%	1
Time to access robotic simulation is a barrier to robotic training	88.89%	11.11%	1
Access to robotic simulation materials is a barrier to robotic training	88.89%	11.11%	1
Credentialing and governance			
Credentialing is important in robotic surgery	88.89%	11.11%	1
A robotic curriculum may inform credentialing	88.89%	11.11%	1
Certification and standardisation of learning			
Evaluation of learners should aim to evaluate for competency (displaying minimum skill to safely accomplish a task)	88.24%	11.76%	2
Evaluation of learners should aim to evaluate for proficiency (consistent demonstration of a high degree of performance)	88.89%	11.11%	1
Video-recording of procedures and video review is an important component of ongoing professional development and learning	100.00%	0.00%	1
A robotic curriculum should be reviewed at least every 3 years to ensure it stays relevant	100.00%	0.00%	1
An international panel of experts should be involved in reviewing the curriculum	94.44%	5.56%	1
Additional consensus statements generated during Delphi round 2			
Robotic surgery training should be available throughout all stages (early, mid and late) of surgical training in a training programme	100.00%	0.00%	2
International robotic surgery curriculum should include considerations in how to set competencies to move from proctored to independent surgery	94.12%	5.88%	2
More research and development is required for procedural simulation (if yes, please elaborate below)	94.12%	5.88%	2
An essential aspect of any curriculum is a methodology for integration into surgical training programs	94.12%	5.88%	2
Robotic proctors should receive appropriate training to adequately deliver robotic training	100.00%	0.00%	2
The curriculum would focus on a structure that is non-platform specific	100.00%	0.00%	2
An international robotic training curriculum should set broad standards but be flexible and adaptable for local and national training environments	94.12%	5.88%	2
Ongoing professional development and refinement of skillsets are required after achieving mastery of basic robotic surgery as outlined by an international curriculum	88.24%	11.76%	2
Supervised training in initial cases on patients is essential after simulation training	100.00%	0.00%	2
Additional consensus statements generated during Delphi round 3			
Ongoing research on multi-platform learning is important	100.00%	0.00%	3
Ongoing and future research should include the transference of skill from one robotic platform to another in an attempt to develop a multiplatform training curriculum	100.00%	0.00%	3
An international robotic curriculum should promote the incorporation of validated metrics to guide and quality assure learning	100.00%	0.00%	3
Other training strategies e.g. off console strategies such as video based learning, cognitive simulation should be adopted to maximise efficiency in training,	92.86%	7.14%	3
Agreed parameters to measure learning curve should be a focus for future work	100.00%	0.00%	3
An international robotic curriculum should include guidance on safe commencement of operating on patients, e.g. endorsing robotic fellowship training, navigating multilevel learning, proctoring, use of appropriate metrics and video review	92.86%	7.14%	3
An international robotic curriculum should be adaptable to multi-platform learning	100.00%	0.00%	3
An international robotic curriculum should broadly endorse the development of procedure specific learning strategies	100.00%	0.00%	3

### Face to face consensus session

A face-to-face consensus session was held at the Society of Robotic Surgeons and consensus was reached on 147 elements in 15 categories including elements added during round 1 and 2. Full results of the final voted on statements that achieved consensus are given in Table 1.

#### Role of an international robotic curriculum

There was a broad consensus on the core role of an international robotic curriculum in setting standards and improving patient safety.

#### Core components of an international robotic curriculum

A stepwise modular multimodal approach was endorsed. Robotic training should span from pre- device learning in basic robotic principles, to simulation- based training and then clinical cases.

#### Flexibility of an international curriculum

Both adaptation over time and flexibility to adapt to the training ecosystem were agreed to be required.

#### Educational goals of an international robotic curriculum

In this section, it was agreed that both technical and non-technical skills should be included. Regardless of the precise definition of competency versus proficiency favoured, having a benchmark to demonstrate skill acquisition and training to a standard was agreed by all to be important. It was agreed that robotic curricular benchmarks should be adaptable to different specialties.

#### Basic didactic pre-device training

Only 7% agreed with the statement that basic didactic pre-device training should be delivered in person, therefore, this statement was removed. The group generally endorsed the concept of online learning, with hybrid options also receiving support.

#### Device training

Multiplatform device training was favoured. Only 17% supported the statement that training should be device specific, so this was removed. Areas that received 100% consensus included the need for docking and undocking to be included, and hands on both console and bedside training.

#### Basic console skills simulation

Multimodal console basic skill simulation was supported, with consensus reached that virtual reality (VR) simulation as well as synthetic or tissue-based box trainer models should be used.

Only 11% agreed with the statement that VR simulators were enough, therefore, it was removed. A modular option to train across platforms was supported.

#### Advanced didactic training

Procedure specific advanced didactic training was endorsed as an important part of a robotic training curriculum.

#### Advanced procedural training

Only 5.5% supported that statement that VR simulation was sufficient for advanced procedural training, so it was removed. High fidelity models were deemed important in advanced procedural training- e.g. synthetic, cadaveric or tissue models. The utilisation of benchmarks was again endorsed, with clinically relevant performance metrics desirable.

#### Models for training

There was unanimous agreement that synthetic models e.g. hydrogels are an acceptable alternative to cadaveric or animal models for basic training. Only 11.11% agreed with the statement that live animal models were essential for training, so it was removed. Simulation models form an important part of the continuum of robotic surgical training; only 17% agreed with the statement that the learner could gain sufficient experience from simulation models alone (i.e. without clinical experience), so this statement was rejected. The ability to dissect, cauterise and replicate procedural steps were seen as important components of robotic surgical training. There was agreement that the exact model used was not as important as the functionality of the model.

#### Operation room training

It was agreed that the preclinical training should include benchmarks to determine if the learner was ready to proceed to operating on humans. The sign off was agreed to be different for someone aiming to do component operating on fellowship compared to someone trying to adopt robotic training in independent practice.

#### Non-technical skills training

Non-technical skills were deemed to be important to be both taught and assessed in a robotic surgical curriculum. These included communication with the entire theatre team.

#### Implementation of an international robotic curriculum

Time, access to robotic training facilities and cost were all agreed to be barriers in delivery of a robotic curriculum.

#### Credentialing and governance

A robotic curriculum was deemed to be helpful for credentialing and governance.

#### Certification and standardisation of learning

Benchmarking was again supported, although again there was debate about the precise definition. Ongoing review of the curriculum to stay up to date was deemed important.

#### Additional comments

One additional comment was rejected which was that a robotic curriculum should only be in the preclinical phase. The stage of training was mentioned in the additional statements added, with 100% consensus achieved that robotic training should be introduced across all stages of training from early to late. Multiplatform training was again endorsed.

### Consensus session thematic analysis

#### Multiplatform training

Discussion centred around challenges and considerations in multiplatform training, and transferability and adaptability of skills across different platforms. Suggestions and solutions to the current challenge of designing multiplatform training included defining the ideal approach to multiplatform training including the different training needs of the platforms. Consideration was raised regarding standardisation of platforms and how this might impact training. Certification and credentialing for individual platforms were discussed with reflection on aviation standards; however, no consensus approach for this was derived from the consensus session.

#### Multilevel learners and adaptive training

Debate regarding this theme highlighted that real life training may need to be adjusted or adapted for trainees at differing levels of experience. A pragmatic approach was suggested making use of available training opportunities to remove barriers to training, however, emphasising benchmarks and clear pathways for progression through training regardless of previous experience. Component operating was discussed as a solution to improving access when training multilevel learners.

#### Competency and proficiency in robotic surgical training

Use of specific training language was discussed and it was determined that the use of concepts of performance benchmarks to guide assessment should be a key component of a robotic surgical curriculum. Methods for defining suggested performance included utilising technology and partnership with industry for large scale data collaboration. A need for a clear definition of competency and proficiency was highlighted as these terms are used inconsistently in education at present.

#### Meaningful assessment and feedback for learning

The use of data, that which will have meaningful impact and be actionable for learners was highlighted as a key concept in training design. It was agreed that using collaborative data sets will assist in assessment design, enhance performance and provide insightful feedback that identifies specific areas for improvement.

#### Bridging the gap between pre-clinical and clinical learning

The key consideration discussed in this theme was ensuring a seamless and fluid transition between clinical and preclinical training and the value of including learners in the clinical environment at all levels to provide real context learning to enhance learning. Further consideration was given to the development of trainers’ curriculum to support this.

#### Ensuring equity and diversity in access to robotic training

It was agreed that addressing the disparity between the perceived importance of training and access was essential to ensure equitable training opportunities. It was unanimously recognised that this requires collaboration between stakeholders and a multifaceted approach including development of novel training technologies.

Thematic analysis and recommendations from the consensus session are included in Table 2.

**Table 2:** Key themes from consensus session analysis

*Multiplatform training*	
Training for a multi-platform world is necessary and requires further dedicated research	
Challenges	“Do we see it as problematic?” “Differentiator is the device training”
Considerations	“Legal responsibility for the vendor in device training” “Debate on basic skills training and industry verification”
Transferability	“Basic simulation skills transferable but dependent on learner” “Need to define differences and potential errors in simulation-based space” “Errors from dissimilar systems interfere with learning and performance”
Curriculum design	“Conformity in controls going forward” “Conformity in basic skills training for quality assurance” “Different systems may require different approaches”
College/regulatory perspective	“Concentration on consultant trainers using their expertise” “Exposure to different platforms for trainees” “Need for postgraduate training body rather than product affiliation”
Industry perspective	“Need for industry to think about standardization in design” “Call for standardization of design to ease the burden on surgeons”
*Multi- level learners and adaptive training*	
Need to develop structured training, with clear benchmarks with adaptable approaches that caters for learners at all stages of their training journey	
Integration of learning	“Training probably isn’t linear” “How do we address the challenge of multi-level learners and make a pragmatic solution for real-life learning?” “Do we think we need to get to the same level of proficiency at entire tasks, at entire procedures?”
Structured training	“Each level needs to go through the same process” “Tailoring assessments to individual learners” “Benchmark-based progression for proficiency”
Challenges	“How do we integrate these concepts into component operating?” “Ensuring access to training without barriers” “Clarifying the role of benchmarks in real-life surgical practice”
Adaptability and individualised learning	“Adapting to different styles of learners and educators” “Modeling training to accommodate different hospital settings” “Tailoring assessments and feedback to individual learner
Leadership in learning	“Standardization of training methodologies” “Leadership in implementing proficiency-based progression” “Utilizing data for scalable and universal training solutions”
*Competency and proficiency in robotic surgical training*	
While there is agreement in the need for benchmarks and standards there are discrepancies in how competency and proficiency are defined in robotic surgical training	
Utilising data	“Just because you can measure that particular data point doesn’t mean it’s going to be helpful in assessment or feedback” “Being able to parse out the top actionable metrics from a large dataset is essential” “We have the opportunity to build novel ways of delivering feedback and metrics using big data sets”
Integration of assessment and feedback	“Assessment and feedback need to be integrated together” “Feedback should be delivered in a way that is meaningful for both the learner and the trainer” “Metrics flow from surgeons who actually can do the procedure, and they provide a standardized approach for assessment and feedback”
Tailored and personalised feedback	“Tailored training programs can be developed based on individual performance metrics” “Metrics allow for the identification of areas of difficulty and proficiency, enabling personalized feedback and training” “Metrics serve as the starting point for providing feedback and enhancing performance”
Assessment metrics	“Metrics for assessment should be prospectively agreed upon to ensure fairness” “Assessment in areas that haven’t been agreed upon at the start should be avoided” “Ongoing international collaboration is needed to develop meaningful metrics using all available data”
*Bridging the gap between pre-clinical and clinical learning*	
Supporting the transition between pre- clinical and clinical learning includes integrating learners and teaching into clinical settings supported by train the trainers programs	
Supporting transitions	“There doesn’t have to be a distinct line between preclinical and clinical learning” “COVID taught us the importance of adapting and transitioning smoothly between preclinical and clinical settings” “The ultimate endpoint is the same for learners, regardless of whether they are in a preclinical or clinical phase”
Integrating learning and learners	“Teaching forces learners to deepen their understanding and pass on clinical knowledge” “Multi-level teaching enhances understanding and facilitates the transfer of clinical knowledge”
Train the trainers to bridge the gap	“Train the trainers programs are crucial for equipping educators with the skills to integrate preclinical learning into the clinical setting” “Train the trainers programs are fundamental for ensuring educators are equipped to train learners effectively”
*Ensuring equity and diversity in access to robotic training*	
Strategies are needed to ensure equity and diversity—this could include VR training, training centres and improving trainee awareness of robotic training opportunities	
Access to robotic training	“Only a small percentage of trainees have access to robotic training despite its perceived importance in future medical practice” “Limited access to robotics training poses a significant barrier to equity and diversity in medical education” “Addressing the gap between perceived importance and actual access to robotic training is crucial for ensuring equitable opportunities for all learners”
Strategies to improve access	“Utilizing VR headsets and centralized training centres can expand access to robotic training” “Raising awareness among learners about the importance of technology in their development can help address disparities in access” “Improving access to robotic training requires a multifaceted approach involving novel technologies and collaboration among stakeholders”

## Discussion

This consensus statement considered the objectives of a robotic curriculum for implementation across diverse training ecosystems. The complexity of the current training landscape in robotic surgical training is immense. Training needs now require consideration of the requirements of learners—which are a diverse group of adult professional learners from all stages of surgical training, and the requirements of robotic surgical practice which now includes the use of novel robotic platforms. This consensus statement includes incorporating international perspectives from a multidisciplinary stakeholder group and addressing the training needs across multi- specialty, multi- level learners and multi-platform environments. The strength in this approach is that the recommendations are adaptable across contemporary training contexts.

Other consensus statements have aimed to address specific aspects of robotic curricula, or specific training contexts. In this Delphi consensus, several novel challenges were identified including training design for multiplatform training, the ideal approach to utilisation of metrics and potential approaches to robotic surgical credentialing. The specific learning needs in multi-platform training have not yet been fully elucidated or the best way to deliver content in this context. Solutions to the multiplatform training needs of contemporary surgical practice are an area which should be the focus of future research effort, however, will require collaboration with industry.

The common barriers to robotic surgical training internationally remain as access and cost. Being strategic in methods of content delivery and using framework map components of training may be one way to promote local access to robotic training. This would be facilitated by recognition of training components internationally across different training contexts. In robotic surgical training there is a general shift away from the use of traditional mandatory animal-based models to more diverse training models including hydrogel models. In discussion of the utility of these models the ability to diathermy, dissecting and training for basic robotic skills was deemed more important than the specific model. The use of hydrogel models was deemed acceptable in this Delphi.

The limitations of the study was that stakeholders included were those attending the Society of Robotic Surgeons for the face-to-face component and able to attend the Delphi session. There was also the potential for bias as several members of the panel represented robotic training academies with the session organised by IMRA and there was representation by ORSI, robotic vendors (CMR) and the Royal Colleges. IMRA also has an arm Pindari that produces hydrogel-based models. Although the range of stakeholders introduces potential bias, it is also useful to consider in a qualitative framework as the experts were situated in their work and were able to contribute to the discussion acknowledging how they were situated. Their unique perspectives were key to this Delphi session.

In conclusion, this Delphi establishes standard principles for robotic training curricula internationally and endorses integration of robotic training to all stages of training, a platform agnostic approach to robotic training and the utilisation of models rather than relying on VR alone in surgical simulation.

## Data Availability

No datasets were generated or analysed during the current study.
